# Circulating Very-Long-Chain Saturated Fatty Acids Were Inversely Associated with Cardiovascular Health: A Prospective Cohort Study and Meta-Analysis

**DOI:** 10.3390/nu12092709

**Published:** 2020-09-04

**Authors:** Meng Liu, Luo-Shi-Yuan Zuo, Ting-Yu Sun, Yan-Yan Wu, Yu-Ping Liu, Fang-Fang Zeng, Yu-Ming Chen

**Affiliations:** 1 Department of Epidemiology, School of Public Health, Sun Yat-Sen University, Guangzhou 510080, China; lium37@mail2.sysu.edu.cn (M.L.); zuolshy@mail2.sysu.edu.cn (L.-S.-Y.Z.); sunty6@mail2.sysu.edu.cn (T.-Y.S.); wuyy53@mail2.sysu.edu.cn (Y.-Y.W.); liuyp57@mail2.sysu.edu.cn (Y.-P.L.); 2 Department of Epidemiology, School of Medicine, Jinan University, No.601 Huangpu Road West, Guangzhou 510630, China; zengffjnu@126.com

**Keywords:** very-long-chain saturated fatty acid, cardiovascular health, cohort study, meta-analysis

## Abstract

Saturated fatty acids with different chain lengths have different biological activities, but little is known about very-long-chain saturated fatty acids (VLCSFAs). This study investigated the associations between the circulating VLCSFAs and cardiovascular health. This community-based cohort study included 2198 adults without carotid artery plaques (CAPs) at baseline. The percentage of baseline erythrocyte VLCSFA (arachidic acid (C20:0), behenic acid (C22:0), and lignoceric acid (C24:0)) was measured by gas chromatography. The presence of CAPs was determined at baseline and every 3 years thereafter by ultrasound examination. A meta-analysis was conducted to summarize the pooled associations between circulating VLCSFAs and the risk of cardiovascular diseases (CVDs). During a median of 7.2 years of follow-up, 573 women (35.1%) and 281 men (49.6%) were identified as CAP incident cases. VLCSFAs were inversely related with CAP risk in women (all *p*-trend <0.05) but not in men. Multivariate adjusted hazard ratios (HRs) and 95% confidence intervals (CIs) of CAPs for the highest (vs. lowest) quartile were 0.80 (0.63–1.01) for C20:0, 0.71 (0.56–0.89) for C22:0, 0.75 (0.59–0.94) for C24:0, and 0.69 (0.55–0.87) for total VLCSFAs in women. The pooled HRs (95% CIs) of CVDs for the highest (vs. lowest) circulating VLCSFAs from seven studies including 8592 participants and 3172 CVD events were 0.67 (0.57–0.79) for C20:0, 0.66 (0.48–0.90) for C22:0, and 0.57 (0.42–0.79) for C24:0, respectively. Our findings suggested that circulating VLCSFAs were inversely associated with cardiovascular health.

## 1. Introduction

Atherosclerosis, a chronic inflammatory disease involving the deposition of cholesterol and foam cell formation within artery walls, is the dominant cause of cardiovascular disease (CVD) [1]. CVD, particularly ischemic heart disease and stroke, was the major cause of death and accounted for 18 million deaths worldwide in 2017 [2]. In China, approximately two in five deaths were caused by CVD in 2014 [3]. Dietary fat has been suggested to play an important role in the development of atherosclerosis and CVD [4].

Circulating fatty acids (FAs), particularly blood cell membrane fatty acids, reflect the quantity and quality of dietary fat intake and influence endogenous lipid metabolism [5]. Saturated fatty acids (SFAs) account for 2.9~20.9% of the total daily energy intake in general populations [6] and represent 30–50% of the total FAs in the human body [7]. Positive associations between the proportions of SFAs, particularly palmitic acid (16:0), with atherosclerosis [8,9] and CVD risks [10,11] have been reported. However, SFAs with different chain lengths exhibit differences in endogenous metabolism [12] and biological activities [13,14]. Previous studies also revealed inverse associations between the proportions of circulating very-long-chain SFAs (VLCSFAs), with 20 carbons or more, and CVD-related risk factors, such as subclinical inflammation [15], adverse metabolic profiles [16], and insulin resistance [17].

Circulating VLCSFAs are derived from limited foods in the diet, such as canola oil, peanuts, and macadamia nuts [18], and are synthesized endogenously from the elongation of 18:0 catalyzed by substrate-specific FA elongases (elovl), such as elovl1 and elovl3 [19]. As an important constituent of sphingolipids, such as ceramides and sphingomyelins, the circulating proportions of VLCSFAs are also influenced by genetic factors related to sphingolipid synthesis [20].

To date, several epidemiological studies have examined the associations between circulating VLCSFA proportions and CVD events (presence, incidence, or death). Beneficial associations of circulating proportions of VLCSFAs, including arachidic acid (C20:0), behenic acid (C22:0), and tetracosanoic acid (C24:0), with the risks of coronary heart disease (CHD) [21,22], heart failure [23], atrial fibrillation [24], sudden cardiac arrest [25], and/or CVD mortality [14] were observed in the Cardiovascular Health Study [14,23,24], the Prevención con Dieta Mediterránea trial [21], the Nurses’ Health Study [22], and the Health Professionals Follow-Up Study [22]. However, no associations were observed in the cohort study from the Ludwigshafen Risk and Cardiovascular Health study [11] and the Physicians’ Health Study [26]. Since the numbers of published studies and participants involved were limited for the association between the proportion of each individual VLCSFA and each CVD endpoint, these associations remained speculative. Moreover, to the authors’ knowledge, no published study has yet evaluated the associations between circulating VLCSFA proportions and atherosclerosis.

We thus examined the associations of erythrocyte proportions of total VLCSFAs and individual VLCSFAs (C20:0, C22:0, and C24:0) with the incidence of atherosclerotic carotid artery plaques (CAPs) in middle-aged and elderly Chinese adults, and we conducted a meta-analysis to summarize the current evidence assessing associations between the proportions of individual VLCSFA and risks of CVD events.

## 2. Materials and Methods

### 2.1. Study Participants

The Guangzhou Nutrition and Health Study (GNHS) is an ongoing community-based prospective cohort study established in 2008 to investigate the determinants of cardiovascular disease and other chronic diseases. Details of the GNHS were described previously [27]. Briefly, 4048 participants aged 40–80 years who have lived in urban Guangzhou for more than 5 years (3169 between 2008 and 2010, and 879 between 2012 and 2013) were recruited by advertisements, health talks, and referrals at baseline. A carotid ultrasound examination was performed to detect CAPs at baseline and every 3 years thereafter. We excluded participants with CAPs (*n* = 824) and participants with hospital-confirmed serious diseases (*n* = 88), such as CHD, stroke, renal failure, senile dementia, and cancer at baseline. Erythrocyte proportions of fatty acids were determined from the baseline blood sample. Subjects without erythrocyte VLCSFAs data (*n* = 354) or without a follow-up ultrasound examination (*n* = 584) were also excluded. A total of 2198 participants were retained in the final analyses, with a median of 7.2 years (interquartile range: 3.9 years) of follow-up.

All participants provided written informed consent at each visit, and the GNHS study had obtained approval for the protocols and procedures from the Medical Ethics Committee of School of Public Health, Sun Yat-sen University (no. 2012-01 and no. 2018-048).

### 2.2. Blood Collection and Erythrocyte FA Measurements

After an overnight fast for more than 10 h, antecubital venous blood samples were collected from the participants and separated (into plasma, leukocytes, erythrocytes, etc.) within 2 h. All biological samples were stored at −80 °C until analysis.

Methods for the assessment of the percentage of erythrocyte FA concentrations have been described previously in detail [28]. Erythrocytes were hemolyzed in hypotonic Tris–HCL buffer (10 mmol/L, pH 7.4, 4 °C) for 2 h. The erythrocyte membrane was collected by ultracentrifugation (12,000 rpm for 30 min at 4 °C) for lipids extraction using chloroform/methanol (2:1, *v/v*) supplemented with 0.005% butylated hydroxytoluene. FAs were trans-methylated with 14% boron-trifluoride ether/methanol (1:3, *v/v*) solution at 100 °C for 5 min. Then, the FA methyl esters (FAMEs) were extracted with hexane, dried under nitrogen, and re-dissolved in hexane for analysis.

FAMEs were analyzed using an Agilent 7890A gas chromatograph (Agilent, Palo Alto, CA, USA). DB-23 capillary column: 60 m × 0.25 mm internal diameter × 0.15 μm film (Agilent, Palo Alto, CA, USA) and by using commercial standards (Nu-Chek Prep). The carrier gas was nitrogen (1.0 mL/min) and the split–splitless injector was used with a split:splitless ratio of 50:1. The injection temperature was 270 °C and detection temperature was 280 °C. The starting temperature of the column was 50 °C and remained for 1 min. The temperature was increased from 50 to 150 °C (25 °C/min), kept 4 min at 150 °C, then from 150 to 180 °C (2 °C/min); 4 min later, the temperature was continuously increased at 3 °C/min up to 220 °C and kept for 24 min.

The individual FAMEs were identified by comparing the retention times with standards. Finally, according to the peak area, the individual erythrocyte FAs were quantified as % of all the fatty acid types detected. The VLCSFAs, as reported by previous studies [21,22], mainly including C20:0, C22:0, and C24:0, were also assessed in this study. The intra-assay coefficients of variation for 42 parallel duplicate samples were 6.38%, 14.37%, and 7.11% for C20:0, C22:0, and C24:0, respectively.

### 2.3. Ultrasound Examination

Participants underwent carotid artery noninvasive B-mode ultrasound (SonoScape SSI-5500, Shenzhen, China) examinations using a high-resolution linear-array transducer system (7.0 to 12.0 MHz) at baseline and every 3 years thereafter. The intima-media thickness (IMT) was manually measured with an electronic caliper, and plaques were defined as a focal IMT greater than 1.5 mm sought in transverse planes (measured in longitudinal planes) based on the Mannheim consensus (2004–2006–2011) [29], regardless of the location on the side of the carotid artery, in any wall of the carotid artery or in any carotid artery segment as follows: (1) common carotid artery (CCA), (2) bifurcation of the carotid artery (BIF), (3) the origin of the internal artery, and (4) the origin of the external artery. Participants with no CAPs at baseline who developed CAPs during the follow-up period were identified as CAP incident cases. Color Doppler exam was performed besides the B-mode to confirm the plaque presence. We also counted the CAPs at the three follow-up visits. Participants with new CAPs or more CAPs than that at the first follow-up visit were identified as CAP progressed cases. In the four ultrasound examinations, a total of nine sonographers participated in the CAP measurement. The intra-observer and inter-observer reliability presented good. Pearson’s correlation coefficients obtained for each sonographer by remeasuring the IMT of 63 randomly selected participants within one hour were 0.979 for CCA and 0.982 for BIF, on remeasuring the IMT of 10 randomly selected participants within three months they were 0.896 for CCA and 0.897 for BIF; coefficients of variation for different sonographers by remeasuring the IMT of 22 randomly selected participants within one hour were 0.853 for CCA and 0.890 for BIF. The sonographer was blinded to the identity and laboratory and clinical information of all participants.

### 2.4. Measurement of Covariates

At baseline, a face-to-face interview was conducted by trained researchers using a structured questionnaire to collect the basic information from participants, such as sociodemographic characteristics, dietary factors, health-related lifestyle factors, self-reported history of diseases, and menopause status. A 19-item 24 h physical activity questionnaire was used to estimate the metabolic equivalent (MET) intensity (sleeping and sitting were excluded). Habitual dietary intake of participants in the previous year were estimated using a validated 79-item quantitative food-frequency questionnaire (FFQ) [30]. Dietary nutrient and energy intakes were calculated by referring to the Chinese Food Composition Table 2004, and the intake of nutrients was adjusted for energy using the residual method [31]. The weight and height of the participants was measured when they were wearing light-weight clothing and no shoes. The body mass index (BMI) was calculated as weight in kilograms divided by height in meters squared.

### 2.5. Statistical Analysis

#### 2.5.1. Erythrocyte VLCSFA Proportions and the Risk of CAP in the GNHS

The statistical analyses were conducted using SPSS software (version 23.0, IBM Corporation, Armonk, NY, USA), and a two-sided *p*-value < 0.05 was considered statistically significant.

Differences in participants’ characteristics between the CAP and non-CAP groups at baseline were compared using *t*-tests or the Mann–Whitney U test for continuous variables and chi-square tests for categorical variables. We also compared the characteristics of subjects included and not-included in this study. We classified participants into sex-specific quartiles according to the proportion of each individual VLCSFA and total proportions of VLCSFAs, respectively, and assumed that the quartiles were continuous variables to calculate the *p*-value for the linear trend (*p*-trend) across increasing quartiles. Separate Cox regression analyses were performed to estimate the hazard ratios (HRs) and 95% confidence intervals (CIs) for the associations between the proportions of total and individual VLCSFAs and the incidence CAP as well as the CAP progression, in women and in men. According to the biological relevance, we adjusted for baseline age in model 1, and further adjusted for education proportion (<9, 9–12, and >12 years), household income (<1500, 1500–3000, and >3000 yuan/month/person), smoking status (yes/no), alcohol consumption (yes/no), BMI (kg/m^2^), hypertension (yes/no), diabetes mellitus (yes/no), physical activity (excluding sleeping and sitting), total energy intake and nut intake, and years since menopause (only in women) at baseline, as well as the use of lipid-lowering drugs (mainly referring to statins) during the whole follow-up period (yes/no), in model 2. Additionally, HRs and 95% CIs corresponding to per standard deviation (SD) increase in VLCSFA proportions were also calculated based on model 2. Interaction terms between VLCSFA quartiles and age, education, household income, and BMI were included in model 2 to assess whether the VLCSFAs–CAP associations would be modified by these variables.

Sensitivity analyses were performed to assess the consistency of the results obtained using model 2. We further adjusted for baseline erythrocyte proportions of total even-chain saturated fatty acids (ECSFAs, including C14:0, C16:0, and C18:0) and total n-3 polyunsaturated fatty acids (n-3 PUFAs, including α-C18:3, C20:3, C20:5, C22:5, and C22:6) (model 2a). Next, we repeated the analyses after excluding participants with an inconsistent diagnosis of CAPs at different visits (model 2b). Finally, we reanalyzed after excluding lipid-lowering drug users, hypertensive and diabetic patients, and smokers at baseline (model 2c).

#### 2.5.2. Meta-Analysis

We included observational studies to examine the associations between the circulating VLCSFA proportions in erythrocyte membrane or plasma phospholipids and fatal and nonfatal CVD events, including CHD, heart failure, atrial fibrillation, sudden cardiac arrest, and stroke, as well as CVD mortality. The ORs or HRs calculated according to the highest (vs. lowest) groups of individual VLCSFA or per SD increase in VLCSFA proportions were pooled separately using the inverse variance model. A detailed description of the methods used for the meta-analysis is provided in the Supplementary Methods.

## 3. Results

### 3.1. Erythrocyte VLCSFA Proportions and the Risk of CAP in the GNHS

Of the 2198 participants (1631 women and 567 men) included in the final analyses, 573 women (35.1%) and 281 men (49.6%) developed CAPs during the median 7.2 years follow-up period. Participants with incident CAPs were more likely to be men. More women with CAPs were diagnosed with hypertension and had a greater BMI and ECSFA proportion, were postmenopausal, tended to use lipid-lowering drugs, and were older but had lower proportions of education, total n-3 PUFA and VLCSFAs (all *p* < 0.05). The men with CAPs were also significantly older and exhibited lower total n-3 PUFA proportions (Table 1). Detailed comparisons of each category and individual fatty acids between CAP and non-CAP subjects are shown Supplemental Table S1.

As shown in Supplemental Table S2, the participants included (vs. not-included) in the final analysis had higher levels of education and physical activities but lower levels of BMI, age and household income; and had a higher proportion of women, but lower proportions of smokers, alcohol drinkers, hypertension, and diabetes.

**Figure 1:**
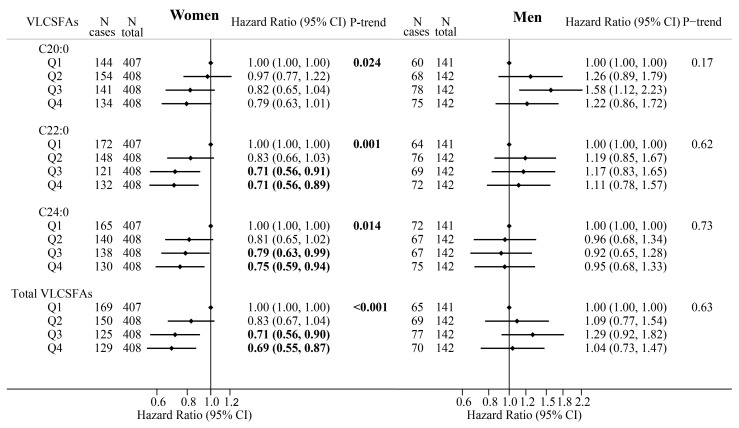
The adjusted hazard ratios (HRs) and 95% confidence intervals (CIs) for carotid artery plaques according to quartiles of the erythrocyte VLCSFA proportions in participants stratified by sex (Women/Men). Abbreviations: Q, quartiles; VLCSFA, very-long-chain saturated fatty acid.

Higher erythrocyte proportions of total and individual VLCSFAs (C22:0, C24:0) were dose-dependently associated with a lower CAP incidence in women (all *p*-trend < 0.05), but not in men (all *p*-trend > 0.05) (Figure 1). Significant sex–VLCSFA interactions for C20:0, C22:0, and total VLCSFAs were observed (all *p* < 0.05). For the age-adjusted model (model 1), the beneficial associations tended to be more significant in women (Supplemental Table S1). The multivariate adjusted HRs (95% CIs) of the highest (vs. lowest) quartile were 0.80 (0.63–1.01) for C20:0, 0.71 (0.56–0.89) for C22:0, 0.75 (0.59–0.94) for C24:0, and 0.69 (0.55–0.87) for total VLCSFAs proportion (model 2) (Figure 1 and Supplemental Table S3).

HRs and 95% CIs were estimated using the Cox regression analysis (the second to fourth quartiles were compared with the first quartile, respectively) and adjusted for age, education level, household income, smoking status, alcohol consumption, body mass index, hypertension, diabetes mellitus, physical activity, dietary nut intake, total energy intake, and years since menopause (women only) at baseline, as well as use of lipid-lowering drugs during the whole follow-up period.

Sensitivity analyses revealed a slight increase in the strength of the beneficial associations after further adjustment for baseline erythrocyte total even-chain saturated fatty acid proportions and total n-3 polyunsaturated fatty acid proportions. The associations were also strengthened after excluding participants with inconsistent CAP diagnoses at three follow-up visits in women. Furthermore, after excluding lipid-lowering drug users, hypertensive and diabetic patients, and smokers at baseline, the beneficial associations remained significant in women (Table 2). In men, a significant positive correlation was observed between the third (vs. lowest) quartile of C20:0 and CAP risk, but no significant associations were found for the proportions of other individual VLCSFA or total VLCSFAs in both model 1 and model 2 (Figure 1 and Supplemental Table S3). HRs and 95% CIs corresponding to per SD increase in VLCSFA proportions are shown in Supplemental Table S4.

Interactions between age and the proportions of total VLCSFAs and C22:0 for the risk of CAPs were observed, but not other demographic and socioeconomic factors, such as educational proportion, household income, and BMI (P for interactions ranged from 0.111 to 0.946, data not shown). However, the interactions between age and VLCSFA proportions were not significant in women (P-interaction ranging from 0.105 to 0.865). Lower erythrocyte proportion of C22:0 was also dose-dependently associated with a faster CAP progression in women (*p*-trend = 0.045) (Supplemental Table S5).

### 3.2. Meta-Analysis

**Figure 2:**
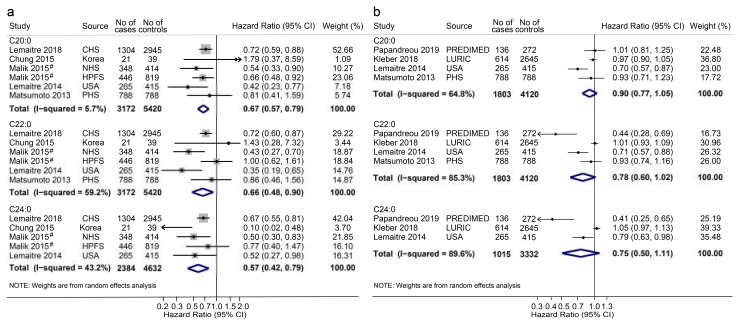
Forest plot of studies investigating the associations between blood very-long-chain fatty acids (VLCSFAs) proportion and cardiovascular diseases. (**a**) Combined hazard ratios (HRs) for the comparison of the highest quantile with the lowest quantile; (**b**) Pooled HRs for per standard deviation (SD) increase. Abbreviations: CI, confidence interval; CHS, the Cardiovascular Health Study; NHS, the Nurses’ Health Study; HPFS, the Health Professionals Follow-Up Study; USA, the United States of America; PHS, the Physicians’ Health Study; PREDIMED, the Prevención con Dieta Mediterránea trial; LURIC, the Ludwigshafen Risk and Cardiovascular Health study. ^#^ Malik (2015): we used the pooled hazard ratios for the association between plasma and erythrocyte VLCSFA proportions and coronary heart diseases from NHS and HPFS cohort reported in 2015 by Malik.

We screened 1011 articles indexed in PubMed, Embase, and Web of Science published from database inception to 18 July 2019 (Supplemental Figure S1). Nine studies (2 case–control [25,32], 3 nested case–control [21,22,26], and 4 cohort studies [11,14,23,24]) that met the selection criteria were finally included in the meta-analysis. OR/HR and 95% CIs were reported in seven studies [14,22,23,24,25,26,32] for the comparison of extreme groups (with 8592 participants and 3172 CVD events), or each SD increase in VLCSFAs in four studies (with 5923 participants and 1803 CVD events) [11,21,25,26]. The characteristics of included studies are depicted in Supplemental Table S6. Using a random-effect model, the pooled HRs and 95% CIs for CVD risk in a comparison of the extreme groups were 0.67 (0.57–0.79) for C20:0, 0.66 (0.48–0.90) for C22:0, and 0.57 (0.42–0.79) for C24:0 (Figure 2a). The corresponding HRs (95% CIs) per SD increase in the proportions of C20:0, C22:0, and C24:0 were 0.90 (0.77–1.05), 0.78 (0.60–1.02), and 0.75 (0.50–1.11), respectively (Figure 2b). As shown in Supplemental Figure S2, the funnel plots were generally symmetric, and the *p*-values for Egger’s tests and Begg’s tests were 0.911 and 0.851 for C20:0, 0.854 and 0.851 for C22:0, and 0.160 and 0.462 for C24:0, suggesting no significant publication bias. Additional details are shown in the Supplementary Materials.

## 4. Discussion

In this community-based prospective study of the middle-aged and elderly Chinese population, both total and individual erythrocyte VLCSFA proportions were inversely related with the CAP incidence in women but not in men. Moreover, in a meta-analysis performed by pooling data from nine observational studies, we first summarized the relationship between the circulating VLCSFA proportions and CVD risk.

A few studies have examined the associations between blood VLCSFA proportions and the risks of CHD [21,22], heart failure [23], atrial fibrillation [24], sudden cardiac arrest [25], and/or CVD mortality [14]. The findings were generally consistent with our study, as inverse associations between total VLCSFA proportions and the risk of CHD incidence were also reported in two nested case–control studies from the Prevención con Dieta Mediterránea trial with 408 participants (136 cases and 272 controls) [21] and the Nurses’ Health Study and Health Professionals Follow-Up Study with 2027 subjects [22]. Regarding the proportions of individual VLCSFAs, beneficial associations were more frequently found for C22:0 and C24:0 [14,21,22,23,24,25] but less frequently for C20:0 [22,23,24,25]. However, no significant associations of erythrocyte proportions of C20:0, C22:0, and C24:0 with CVD mortality (*n* = 614) were observed in the Ludwigshafen Risk and Cardiovascular Health study with 3259 participants after 9.9 years follow-up [11], as well as in the Physicians’ Health Study for the associations of plasma C20:0 and C22:0 proportions and heart failure [26]. Previous studies also revealed inverse relations between circulating VLCSFA proportions and CVD-related risk factors, such as type 2 diabetes [33], insulin resistance [17], subclinical inflammation [17], and an adverse metabolic profile [16]. Based on findings from previous studies and the current investigation, circulating VLCSFA proportions were beneficially associated with atherosclerosis, CVDs, and CVD-related risk factors.

We also conducted a meta-analysis to summarize the associations between VLCSFA proportions and the risk of CVDs. The results from this pooled analysis indicate that higher percentages of the circulating VLCSFAs C20:0, C22:0, and C24:0 were associated with 23%, 27%, and 31% lower risks of CVDs, respectively (all *p* < 0.05). The original studies were mainly prospective studies [11,14,21,22,23,24], with the exception of two small case–control studies [25,32], and had mean quality score of 7.1 (6–8), as assessed using the Newcastle–Ottawa Quality Assessment Scale. Almost all studies adjusted for covariates. The results of this meta-analysis further supported our findings obtained from this Chinese population.

In some cell culture studies and animal experiments, very-long-chain ceramides also play a protective role in liver homeostasis [34], hepatic steatosis [35], myelin maintenance [36], apoptosis [13], and insulin resistance [35,37] by influencing cell membrane properties and cell signaling. These mechanisms might partially explain the VLCSFA proportions protectively associated with atherosclerosis and atherosclerosis-related diseases. Additionally, VLCSFA proportions were also inversely correlated with an adverse blood lipid profile [16,22], subclinical inflammation [15,22], and insulin resistance [17,22] in epidemiological studies. However, the major sources and biological functions of VLCSFAs are largely unknown; therefore, additional studies are needed to obtain a better understanding of the mechanism underlying the associations between VLCSFA proportions, CVD, and CVD-related diseases.

The beneficial associations were only significant in women but not in men. The nested case–control study from the Nurses’ Health Study and Health Professionals Follow-Up Study [22] also revealed more pronounced inverse associations between total VLCSFA proportions and CHD in women than in men (HR for the highest quintile compared with the lowest quintile: 0.39 vs. 0.89 in erythrocytes; 0.37 vs. 0.57 in plasma). Unfortunately, results stratified according to sex were not available in most of the other studies. Erythrocyte VLCSFAs were positively correlated with plasma ferritin proportions in Chinese men, but not in women, in a previous study [38]. The “iron-heart hypothesis” might partially explain the sex difference. Moreover, women might be more sensitive to fatty acids than men are [39]. The explanations for the different results in the two sexes remain largely unclear. Further studies are needed to clarify this issue.

This study possessed many strengths, as described below. First, to the authors’ knowledge, this prospective study is the first to report beneficial associations between circulating proportions of the VLCSFAs C20:0, C22:0, and C24:0 and the incidence of carotid artery plaques. Our findings provided further evidence supporting the inverse associations between VLCSFA proportions and CVDs via anti-atherosclerotic activities, since artery atherosclerosis is a pathological change and indicator of CVDs at an earlier stage. Second, we observed a consistent result for the proportions of C20:0, C22:0, and C24:0 alone, as well as the total VLCSFAs proportion, and for a series of sensitivity analyses that refuted the possibility that the positive associations occurred by chance. Third, our findings were replicated in the meta-analysis of the VLCSFA-CVD associations.

Some limitations merit consideration. First, we were unable to verify the causal relationship between VLCSFA proportions and CAP due to the inherent weakness of observational studies, although the prospective study design might strengthen the temporal sequence. Next, erythrocyte VLCSFA proportions were only determined at baseline. However, the blood cell membrane FA proportions reflect habitual diet intake (2–3 months) and are relatively stable [5]. Although VLCSFA proportions might vary over time, this source of measurement error would have biased results toward the null hypothesis. Another limitation of using erythrocyte fatty acids as the internal exposure was that the fatty acids in red blood cell membrane were more likely to be affected by the body metabolic features than those in plasma. Third, potential misclassifications of participants with CAP whose IMT was approximately 1.5 mm might occur. As predicted, the beneficial associations were more pronounced after excluding participants with differences noted in the CAP classification at follow-up visits. Fourthly, we only analyzed the association between the VLCSFAs and the incidence of CAPs, without any other plaque burden outcome. However, we counted CAPs at the follow-up visits, and the C22:0 was still inversely associated with the progression of CAPs in women. Fifthly, a number of participants were not included in the final analysis, and as predicted, they had more unhealth lifestyles and worse physical condition. The loss of this extreme group may underestimate the observed association. Finally, the power was insufficient to determine the VLCSFA–CAP associations in men due to the limited sample size.

## 5. Conclusions

In conclusion, higher erythrocyte proportions of the VLCSFAs C22:0, C24:0, and total VLCSFAs were each associated with a lower CAP incidence in women but not in men. The results of the meta-analysis suggested beneficial associations of circulating VLCSFA proportions with the risk of CVD events. The findings support the hypothesis that higher percentages of VLCSFAs are associated with positive cardiovascular health outcomes.

## Figures and Tables

**Table 1:** Baseline characteristics of a cohort of 2198 Chinese adults with and without carotid artery plaque (CAP) stratified by sex ^a^.

	Men			Women		
	CAP Cases	Non-CAP Cases	*p*-Value ^b^	CAP Cases	Non-CAP Cases	*p*-Value ^b^
N, (%)	281 (49.6)	286 (50.4)		573 (35.1)	1058 (64.9)	
Age, years	59.6 ± 5.9	58.5 ± 5.9	0.026	57.9± 5.0	56.3 ± 5.0	<0.001
BMI, kg/m^2^	23.7 ± 3.0	23.3 ± 2.9	0.090	23.3± 3.2	22.8 ± 3.0	0.001
Education proportion, years, N (%)			0.517			0.001
<9	75 (26.7)	68 (23.8)		189 (33.0)	260 (24.6)	
9–12	108 (38.4)	123 (43.0)		272 (47.5)	570 (53.9)	
>12	98 (34.9)	95 (33.2)		112 (19.5)	228 (21.6)	
Household income, Yuan/month/person, N (%)			0.287			0.726
≤1500	79 (28.1)	74 (25.9)		175 (30.5)	309 (29.2)	
1501–3000	135 (48.0)	127 (44.4)		277 (48.3)	509 (48.1)	
>3000	67 (23.8)	85 (29.7)		121 (21.1)	240 (22.7)	
Smokers, N (%) ^c^	137 (48.8)	119 (41.6)	0.087	2 (0.3)	1 (0.1)	0.252
Alcohol drinkers, N (%) ^d^	45 (16.0)	36 (12.6)	0.244	16 (2.8)	20 (1.9)	0.237
Hypertension, N (%)	58 (20.6)	60 (21.0)	0.921	125 (21.8)	176 (16.6)	0.010
Diabetes, N (%)	9 (3.2)	17 (5.9)	0.119	22 (3.8)	25 (2.4)	0.089
Lipid-lowering drug users, N (%) ^e^	43 (15.3)	39 (13.6)	0.573	148 (25.8)	182 (17.2)	<0.001
Physical activities, MET-hours/day ^f^	25.4 ± 7.0	26.2 ± 7.2	0.200	26.1 ± 6.4	26.5 ± 6.7	0.310
Total energy intake, kcal/day	1986 ± 571	2025 ± 754	0.495	1730 ± 577	1741 ± 609	0.737
Nut intake, g/day	9.4 (15.3)	10.0 (16.4)	0.496	9.6 (16.2)	9.8 (16.2)	0.727
Total n-3 PUFAs, %	6.8 (2.3)	7.1 (2.0)	0.018	7.1 (2.5)	7.2 (2.1)	0.009
Total ECSFAs, %	44.3 (4.5)	44.3 (4.9)	0.639	44.9 (6.3)	44.1 (5.4)	<0.001
Total VLCSFAs, %	6.6 (1.8)	6.5 (1.9)	0.336	6.7 (2.0)	7.0 (2.0)	0.001
C20:0, %	0.42 (0.10)	0.40 (0.10)	0.044	0.44 (0.12)	0.44 (0.13)	0.257
C22:0, %	1.6 (0.7)	1.6 (1.0)	0.470	1.6 (0.7)	1.7 (0.5)	0.001
C24:0, %	4.8 (1.2)	4.7 (1.1)	0.555	4.8 (1.3)	4.9 (1.3)	0.009
Post-menopause women, N (%)	-	-	-	541 (94.4)	943(89.1)	<0.001
Years since menopause, years	-	-	-	7.2 (8.1)	5.5 (7.9)	<0.001

Abbreviations: CAP, carotid artery plaque; BMI: body mass index; n-3 PUFAs: n-3 polyunsaturated fatty acids (α-C18:3, C20:3, C20:5, C22:5, and C22:6); ECSFAs: even-chain saturated fatty acids (C14:0, C16:0, and C18:0); VLCSFAs: very-long-chain saturated fatty acids. ^a^ The data are presented as the mean ± SD or median (interquartile range) according to the distribution of variables. ^b^ *p*-Value for comparisons of participants with plaques to participants without plaques. The chi-square tests were used to analyze categorical variables; *t*-tests were used to analyze normally distributed variables, whereas the Mann–Whitney U-test was used when variables displayed a non-normal distribution. ^c^ Smokers: Those who smoke ≥1 cigarette/day in the past year. ^d^ Alcohol drinkers: Those who drink alcohol ≥1 cup/week in the last year. ^e^ Participants who used lipid-lowering drugs (mainly referring to statins) throughout the follow-up period were lipid-lowering drug users. ^f^ Physical activities, excluding time spent sleeping and sedentary sitting, are presented as metabolic equivalent (MET) hours per day.

**Table 2:** Results of the sensitivity analysis for the associations between the erythrocyte VLCSFA proportions and incidence of carotid artery plaques in women ^a^.

	Quartiles of Erythrocyte VLCSFAs				*p*-Trend ^b^
	Q1	Q2	Q3	Q4	
Total VLCSFA					
Model 2a	1.00	0.88 (0.70–1.10)	0.77 (0.60–0.98) *	0.71 (0.56–0.91) **	0.003
Model 2b	1.00	0.84 (0.67–1.07)	0.71 (0.55–0.92) **	0.66 (0.51–0.85) **	0.001
Model 2c	1.00	0.89 (0.66–1.21)	0.75 (0.55–1.02)	0.69 (0.51–0.95) *	0.012
C20:0					
Model 2a	1.00	0.96 (0.77–1.21)	0.79 (0.62–1.00) *	0.71 (0.56–0.91) **	0.002
Model 2b	1.00	0.98 (0.77–1.25)	0.79 (0.61–1.01)	0.73 (0.57–0.95) *	0.005
Model 2c	1.00	0.98 (0.72–1.33)	0.92 (0.68–1.25)	0.81 (0.59–1.11)	0.167
C22:0					
Model 2a	1.00	0.87 (0.69–1.10)	0.78 (0.60–1.01)	0.72 (0.56–0.94) *	0.012
Model 2b	1.00	0.84 (0.66–1.07)	0.70 (0.54–0.91) **	0.68 (0.53–0.88) **	0.001
Model 2c	1.00	0.86 (0.63–1.16)	0.68 (0.50–0.93) *	0.71 (0.53–0.97) *	0.014
C24:0					
Model 2a	1.00	0.88 (0.70–1.11)	0.86 (0.68–1.09)	0.79 (0.62–0.99) *	0.048
Model 2b	1.00	0.79 (0.62–1.02)	0.81 (0.63–1.03)	0.73 (0.57–0.94) *	0.020
Model 2c	1.00	0.89 (0.66–1.21)	0.78 (0.57–1.06)	0.77 (0.57–1.05)	0.066

Abbreviations: Q, quartile; VLCSFAs, very-long-chain saturated fatty acids. ^a^ Cox regression analysis. Model 2a was further adjusted for baseline erythrocyte total even-chain saturated fatty acid (C14:0, C16:0, and C18:0) and total n-3 polyunsaturated fatty acid (α-C18:3, C20:3, C20:5, C22:5, and C22:6) proportions based on model 2. In model 2b, analyses were repeated based on model 2 after the exclusion of participants with an inconsistent CAP diagnosis at the three follow-up visits (N = 1549 women). In model 2c, we reanalyzed based on model 2 after excluding hypertensive and diabetic patients and smokers at baseline and lipid-lowering drug users during the whole follow-up period (N = 1287). ^b^ Linear trends across increasing quartiles were tested by assuming that the quartiles were continuous variables. Compared with quartile 1: * *p* < 0.05; ** *p* < 0.01.

## Supplementary Materials

The following are available online at https://www.mdpi.com/2072-6643/12/9/2709/s1. Additional Expanded Methods used for the meta-analysis. Figure S1. Flowchart of the study selection process for a meta-analysis of the association between circulating very-long-chain saturated fatty acids and cardiovascular diseases. Figure S2. Funnel plots with pseudo 95% confidence limits of studies that investigated the association between very-long-chain saturated fatty acids and the risk of cardiovascular diseases. Table S1. Erythrocyte membrane fatty acid of Chinese adults from the Guangzhou Nutrition and Health Study (GNHS) with and without CAP stratified by sex. Table S2. The comparison of basic characteristics between subjects included and not-included in the final study. Table S3. Hazards ratios (95% confidence interval) for carotid artery plaques according to quartiles of the erythrocyte VLCSFA proportions stratified by gender. Table S4. Hazards ratios (HR) and 95% confidence interval (CI) for per standard deviation (SD) increase of the erythrocyte VLCSFA proportions. Table S5. Hazards ratios (95% confidence interval) for carotid artery plaques according to quartiles of the erythrocyte VLCSFA proportions stratified by gender. Table S6. Characteristics of the eligible studies.
