# Supplementary material for: Circulating Very-Long-Chain Saturated Fatty Acids Were Inversely Associated with Cardiovascular Health: A Prospective Cohort Study and Meta-Analysis
Source: Nutrients. 2020 Sep 4;12(9):2709. doi: 10.3390/nu12092709 (PMC7551797; doi:10.3390/nu12092709)
Supplement: Supplementary file 1 [file nutrients-12-02709-s001.pdf]

SUPPLEMENTARY MATERIAL

Circulating very-long-chain saturated fatty acids were inversely associated with cardiovascular health: a prospective cohort study and meta-analysis

Meng Liu, Luo-Shi-Yuan Zuo, Ting-Yu Sun, Yan-Yan Wu, Yu-Ping Liu, Fang-Fang Zeng, Yu-Ming Chen.

Additional Expanded Methods used for the meta-analysis

Search strategy

We systematically searched the literature in the PubMed, Embase and Web of Science databases from inception to 18 July 2019. The following search terms based on Medical Subject Headings (MeSH) were used: 1) saturated AND fatty acid* AND very-long-chain; 2) ("C20:0" OR arachidic OR eicosanoic OR "C22:0" OR docosanoic OR behenic OR "C24:0" OR lignoceric OR tetracosanoic) AND acid*; 3) cardiovascular disease OR cardiovascular OR cardi* OR coronary OR heart OR artery OR myocardial OR angina OR brain OR cerebrovascular OR stroke* OR ischemi* OR infarction* OR attack* OR arteriosclerosis OR atherosclerosis OR plaque* OR "carotid obstruction" OR "carotid stenosis" OR "intima-media thickness" OR "arterial wall thickness" OR atherothrombosis; 4) mortality* OR death*. Finally, we combined the aforementioned terms by the strategy as follows: [1) OR 2)] AND [3) OR 4)]. We did not set filters to limit the article types, languages, species or other parameters. In addition, we also manually searched the reference lists of identified articles and reviews to find additional relevant studies missed in the initial research.

Study selection

Eligible studies included in our meta-analysis were required to satisfy the following criteria: 1) original studies that investigated the association between circulating very-long-chain fatty acid (VLCSFA) proportions and (cardiovascular disease) CVD events or CVD mortality; 2) designed as cross-sectional studies, case-control studies and prospective studies, including cohort studies and nested case-control studies; 3) used CVD events (CVD presence, incidence or death) as outcomes; and 4) odds ratios (ORs), relative risks (RRs) or hazard ratios (HRs) with 95% confidence intervals (CIs) were available. We excluded the studies that 1) did not assess the association of interest between VLCSFA proportions and CVD; 2) used dietary VLCSFA proportions or VLCSFA patterns as exposures; or 3) were conference abstracts or editorials. Additionally, if multiple studies from the same cohort with overlapping patient populations were published, we pooled the effect estimates, included ORs, RRs and HRs, of these studies before conducting the meta-analysis.

Data extraction and quality assessment

The following information was independently extracted from the included articles by two investigators: the first author's last name, publication year, study design, study name, duration of follow-up, sample size, number of patients, participants' characteristics (age and sex distributions), assessment of VLCSFA proportions, main outcomes, exposure categories, adjusted confounders, maximum-adjusted ORs/RRs/HRs with corresponding 95% CIs.

Two investigators independently assessed the quality of eligible articles using the Newcastle-Ottawa Quality Assessment Scale. The scale allocates stars based on the quality of three parts, selection, comparability and outcome/exposure, and assessed the quality of studies with a score ranging from 1 to 9 stars. Studies that scored 8-9 stars were assessed as high-quality studies, 4-7 stars as moderate-quality studies and 0-3 as low-quality studies.

If the two investigators had any disagreement during data extraction or quality assessment, discussions with a third investigator were needed to reach a consensus.

Data analysis

The adjusted effect estimates and 95% CIs of included studies were pooled using a meta-analysis and visualized by constructing forest plots. The heterogeneity across eligible articles was

evaluated using the Cochran Q test and I^2 statistics. A random-effect model was selected whether heterogeneity was significant or not. When more than 10 studies were included, the potential publication bias was assessed using funnel plots and quantified using Egger's and Begg's test. All statistical analyses were performed using STATA version 14.0 software (Stata Corp, College Station, TX, USA). A P-value <0.05 was considered statistically significant, unless indicated otherwise.

Results

A flowchart of the study selection process was shown in **Supplemental Figure 1**. After a strict screen based on inclusion and exclusion criteria, 9 studies (2 case-control, 3 nested case-control and 4 cohort studies) were finally included in the meta-analysis. OR/HR and 95% CIs were reported in seven studies for the comparison of extreme groups (with 8592 participants and 3172 CVD events), or each SD increase in VLCSFAs in four studies (with 5923 participants and 1803 CVD events). The main characteristics and quality assessment of the included studies were depicted in **Supplemental Table 4**. Since the subjects in the three studies from the CHS were largely overlapped (heart failure [1] and CHD mortality [2]), we included that one with larger case number (heart failure [1]) in the estimation of pooled the effect. We excluded the results from the CHS using the end-point of atrial fibrillation [3], because the end-point are from the same population, overlapped with heat failure and had weak relationship to atherosclerosis. Additionally, the study in 2015 by Malik et al. reported the associations of CHD with VLCSFA proportions in two independent cohorts (the Nurses' Health Study and the Health Professionals Follow-Up Study); therefore, we also treated this study as two independent studies in the meta-analysis.

The pooled HRs and 95% CIs for the comparison of the highest quantile with the lowest quantile using a random-effect model (the values of I^2 were between 5.7% and 59.2%) were 0.67 (0.57-0.79) for C20:0, 0.66 (0.48-0.90) for C22:0 and 0.57 (0.42-0.79) for C24:0 (**Figure 2a**). And the sensitivity analysis that treated the three CHS studies in the meta-analysis as independent cohorts showed a stable result. However, for each SD increase in C20:0, C22:0 and C24:0 proportions, the combined HRs and 95% CIs obtained using a random-effect model (the values of I^2 were all greater than 60%) were 0.90 (0.77-1.05), 0.78 (0.60-1.02) and 0.75 (0.50-1.11), respectively (**Figure 2b**).

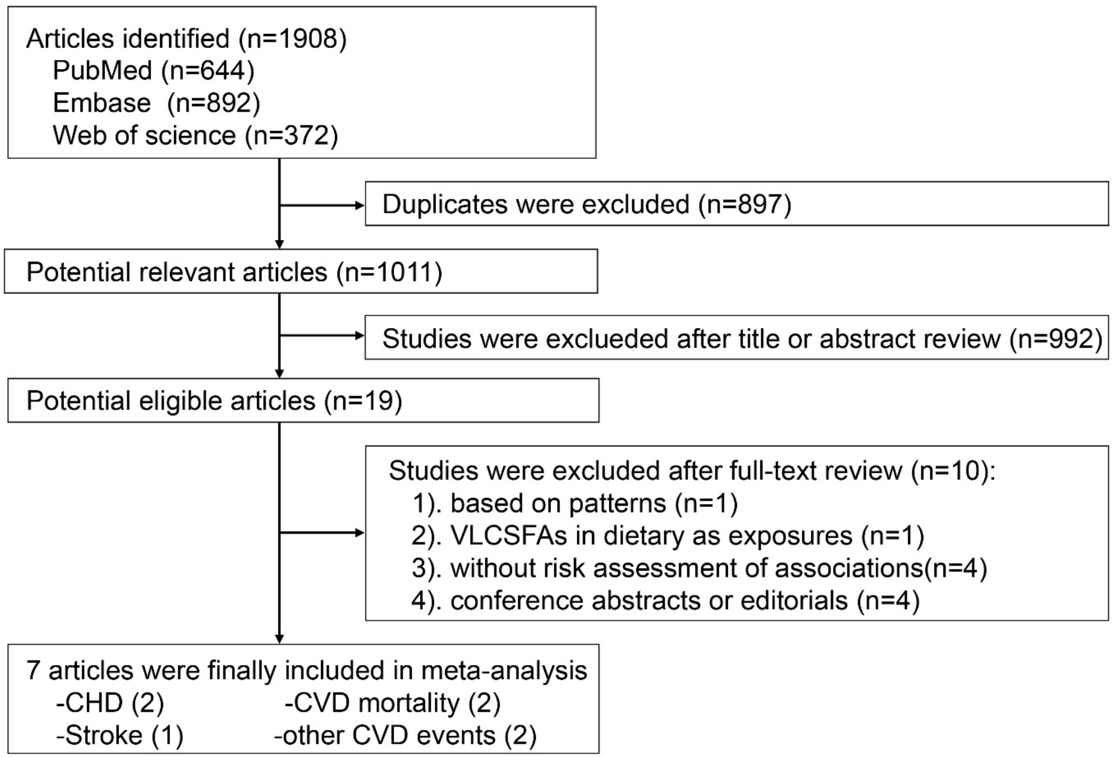

Supplemental Figure S1. Flowchart of the study selection process for the meta-analysis of the association between circulating very-long-chain saturated fatty acids and cardiovascular diseases.

Abbreviations: VLCSFA, very-long-chain saturated fatty acid; CHD, coronary heart diseases; CVD, cardiovascular disease.

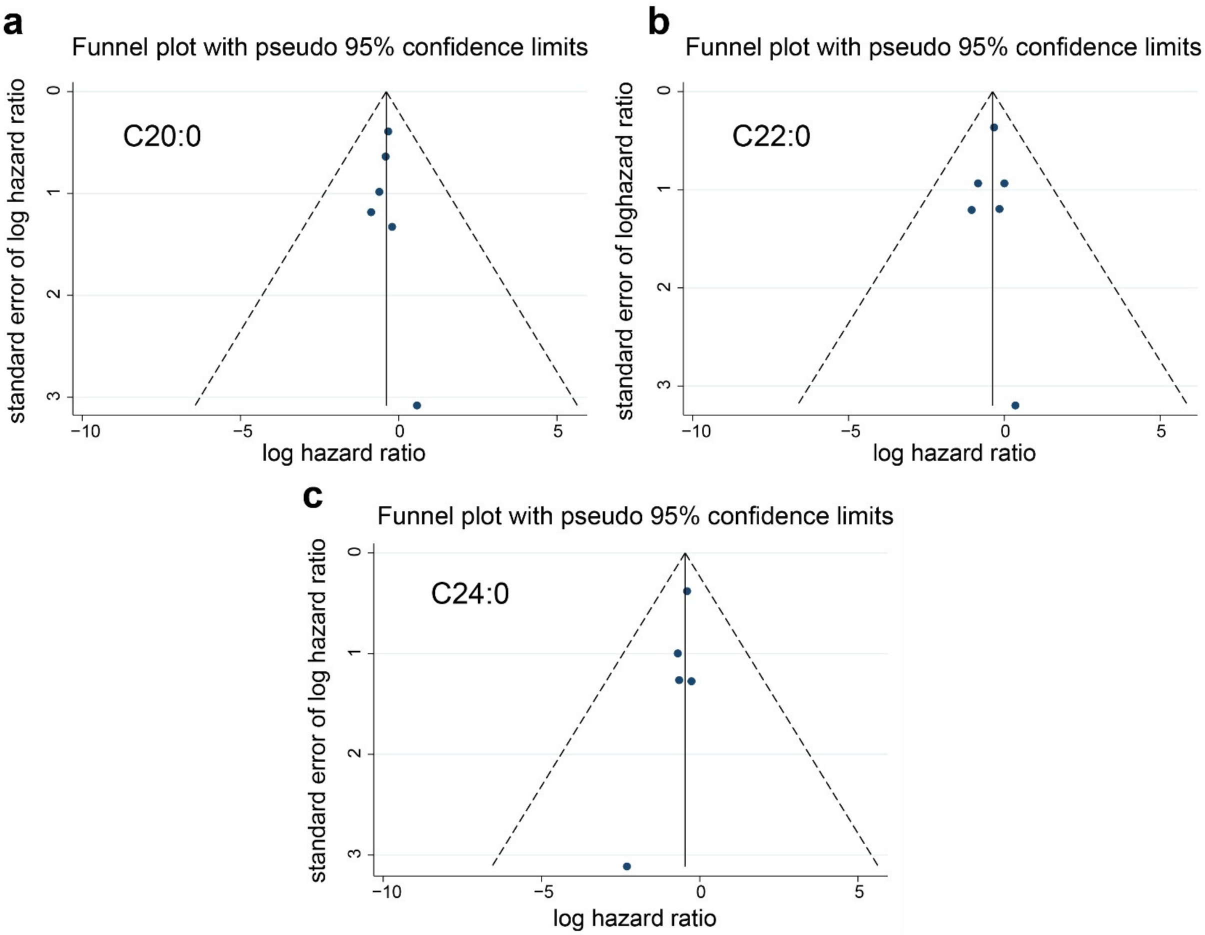

Supplemental Figure S2. Funnel plots with pseudo 95 % confidence limits of studies that investigated the association between very-long-chain saturated fatty acids and the risk of cardiovascular diseases.

Supplemental Table S1. Erythrocyte membrane fatty acid of Chinese adults from the Guangzhou Nutrition and Health Study (GNHS) with and without CAP stratified by sex [a]

	Men			Women		
	CAP cases	Non-CAP cases	P-value [b]	CAP cases	Non-CAP cases	P-value [b]
Total ECSFAs, %	44.3 (4.5)	44.3 (4.9)	0.639	44.9 (6.3)	44.1 (5.4)	**<0.001**
C14:0, %	0.26 (0.09)	0.24 (0.09)	0.097	0.28 (0.12)	0.26 (0.10)	**0.008**
C16:0, %	27.6 (4.1)	27.5 (3.9)	0.730	27.7 (5.0)	27.0 (4.5)	**<0.001**
C18:0, %	16.6 (2.1)	16.6 (1.8)	0.514	17.0 (2.4)	16.9 (2.1)	**0.022**
Total VLCSFAs, %	6.6 (1.8)	6.5 (1.9)	0.336	6.7 (2.0)	7.0 (2.0)	**0.001**
C20:0, %	0.42 (0.10)	0.40 (0.10)	**0.044**	0.44 (0.12)	0.44 (0.13)	0.257
C22:0, %	1.6 (0.7)	1.6 (1.0)	0.470	1.6 (0.7)	1.7 (0.5)	**0.001**
C24:0, %	4.8 (1.2)	4.7 (1.1)	0.555	4.8 (1.3)	4.9 (1.3)	**0.009**
Total MUFAs, %	17.4 (2.1)	17.5 (2.1)	0.453	17.2 (2.0)	17.1 (2.2)	0.755
C16:1, %	0.24 (0.18)	0.26 (0.18)	0.127	0.23 (0.16)	0.24 (0.16)	0.740
C18:1, %	12.2 (1.9)	12.2 (1.8)	0.903	12.1 (1.9)	12.0 (1.7)	0.313
C20:1, %	0.24 (0.06)	0.24 (0.06)	0.787	0.23 (0.07)	0.24 (0.07)	0.280
C22:1, %	0.24 (0.26)	0.28 (0.29)	**0.012**	0.27 (0.27)	0.29 (0.26)	0.326
C24:1, %	4.1 (0.9)	4.1 (0.8)	0.251	4.0 (0.9)	4.1 (0.8)	**0.007**
Total n-3 PUFAs, %	6.8 (2.3)	7.1 (2.0)	**0.018**	7.1 (2.5)	7.2 (2.1)	**0.009**
α-C18:3, %	0.08 (0.04)	0.08 (0.03)	0.241	0.09 (0.04)	0.09 (0.04)	0.276
C20:3, %	0.04 (0.03)	0.04 (0.03)	0.129	0.04 (0.03)	0.04 (0.03)	0.758
C20:5, %	0.53 (0.86)	0.56 (1.02)	0.137	0.54 (0.86)	0.56 (0.51)	0.916
C22:5, %	1.5 (0.5)	1.6 (0.4)	0.056	1.5 (0.5)	1.6 (0.4)	**0.018**
C22:6, %	4.3 (1.7)	4.6 (1.4)	**0.019**	4.5 (1.8)	4.8 (1.6)	**<0.001**
Total n-6 PUFAs, %	24.5 (3.8)	24.6 (3.6)	0.900	24.1 (5.1)	24.7 (3.9)	**<0.001**
C18:2, %	9.9 (2.0)	10.0 (1.8)	0.610	9.7 (2.0)	9.8 (1.8)	**0.015**
γ-C18:3, %	0.04 (0.02)	0.03 (0.02)	**0.015**	0.04 (0.03)	0.04 (0.03)	**<0.001**
C20:2, %	0.37 (0.13)	0.36 (0.13)	0.326	0.35 (0.13)	0.36 (0.11)	0.512
C20:4, %	11.5 (2.5)	11.6 (2.3)	0.323	11.3 (3.3)	11.8 (2.6)	**<0.001**
C22:4, %	2.3 (0.8)	2.3 (0.7)	0.684	2.1 (0.8)	2.2 (0.7)	**0.002**

Abbreviations: CAP, carotid artery plaque; ECSFAs: even-chain saturated fatty acids; VLCSFAs: very-long-chain saturated fatty acids; MUFAs: monounsaturated fatty acids; n-3 PUFAs: n-3 polyunsaturated fatty acids; n-6 PUFA: n-6 polyunsaturated fatty acids.

[a] The data are presented as the median (interquartile range), and the Mann-Whitney U-test was used to compare the participants with plaques to participants without plaques.

Supplemental Table S2. The comparison of basic characteristics between subjects included and non-included in the final study [a]

	Included in the final study	Not-included in the final study	P-value [b]
	N=2198	N=1850	
Age, year	57.4± 5.4	59.9 ± 6.9	**<0.001**
Men, N (%)	567 (25.8)	723 (39.1)	**<0.001**
BMI, kg/m^2	23.1± 3.0	23.7 ± 3.1	**<0.001**
Education proportion, year, N (%)			**<0.001**
<9	592 (26.9)	633 (34.2)	
9-12	1073 (48.8)	755 (40.8)	
>12	533 (24.2)	462 (25.0)	
Household income, Yuan/month/person, N (%)			**<0.001**
≤1500	637 (29.0)	509 (27.5)	
1501-3000	1048 (47.7)	797 (43.1)	
>3000	513 (23.3)	544 (29.4)	
Smokers, N (%) [c]	259 (11.8)	418 (22.6)	**<0.001**
Alcohol drinkers, N (%) [d]	117 (5.3)	165 (8.9)	**<0.001**
Hypertension, N (%)	419 (19.1)	600 (32.4)	**<0.001**
Diabetes, N (%)	115 (5.2)	210 (11.4)	**<0.001**
Lipid-lowering medication users, N (%) [e]	412 (18.7)	309 (16.7)	0.088
Physical activities, MET-hours/day [f]	26.2 ± 6.8	25.0 ± 7.0	**<0.001**
Total energy intake, kcal/day	1806 ± 628	1846 ± 569	**0.029**
Nut intake, g/day	9.7 (16.2)	9.7 (16.4)	0.442
Post-menopause women, N (%)	1484 (91.0)	916 (81.3)	**<0.001**
Years since menopause, year	6.1 (8.1)	6.9 (11.2)	0.199

Abbreviations: BMI: body mass index.

[a] The data are presented as the mean ± SD or median (interquartile range) according to the distribution of variables.

[b] P-value for comparisons of participants with plaques to participants without plaques. The Chi-square tests was used to analyze categorical variables; t-tests were used to analyze normally distributed variables, whereas the Mann-Whitney U-test was used when variables displayed a non-normal distribution.

[c] Smokers: Those who smoke ≥ 1 cigarette/d in the past year.

[d] Alcohol drinkers: Those who drink alcohol ≥ 1 cup/week in the last year.

[e] Participants who used lipid-lowering drugs (mainly referring to statins) throughout the follow-up period were lipid-lowering drugs users.

[f] Physical activities, excluding time spent sleeping and sedentary sitting, are presented as metabolic equivalent (MET) hours per day.

Supplemental Table S3. Hazards ratios (95% confidence interval) for carotid artery plaques according to quartiles of the erythrocyte VLCSFA proportions stratified by gender [a]

	Quartiles by erythrocyte VLCSFA proportions				P-trend [b]
	Q1	Q2	Q3	Q4	
**Women (n=1631)**					
Total VLCSFA					
Median, %	5.26	6.38	7.34	8.83	
Cases/n	169/407	150/408	125/408	129/408	
Model 1	1.00	0.83 (0.66-1.03)	**0.68 (0.54-0.86)** **	**0.67 (0.53-0.84)** **	**<0.001**
Model 2	1.00	0.83 (0.67-1.04)	**0.71 (0.56-0.90)** **	**0.69 (0.55-0.87)** **	**0.001**
C20:0					
Median, %	0.37	0.42	0.47	0.62	
Cases/n	144/407	154/408	141/408	134/408	
Model 1	1.00	0.97 (0.77-1.22)	0.83 (0.66-1.05)	**0.79 (0.62-1.00)** *	**0.021**
Model 2	1.00	0.97 (0.77-1.22)	0.82 (0.65-1.04)	0.80 (0.63-1.01)	**0.024**
C22:0					
Median, %	0.54	1.53	1.78	2.13	
Cases/n	172/407	148/408	121/408	132/408	
Model 1	1.00	0.82 (0.65-1.02)	**0.67 (0.53-0.85)** **	**0.67 (0.54-0.85)** **	**<0.001**
Model 2	1.00	0.83 (0.66-1.03)	**0.71 (0.56-0.91)** **	**0.71 (0.56-0.89)** **	**0.002**
C24:0					
Median, %	3.89	4.57	5.16	6.27	
Cases/n	165/407	140/408	138/408	130/408	
Model 1	1.00	**0.80 (0.64-1.00)** ‡	**0.78 (0.62-0.97)** *	**0.72 (0.57-0.91)** **	**0.006**
Model 2	1.00	0.82 (0.65-1.02)	**0.79 (0.63-0.99)** *	**0.75 (0.59-0.94)** *	**0.014**
**Men (n=567)**					
Total VLCSFA					
Median, %	5.17	6.18	7.01	8.43	
Cases/n	65/141	69/142	77/142	70/142	
Model 1	1.00	1.09 (0.78-1.54)	1.22 (0.88-1.70)	1.02 (0.73-1.44)	0.758
Model 2	1.00	1.09 (0.77-1.54)	1.29 (0.92-1.82)	1.04 (0.73-1.47)	0.634
C20:0					
Median, %	0.35	0.39	0.44	0.55	
Cases/n	60/141	68/142	78/142	75/142	
Model 1	1.00	1.28 (0.91-1.82)	**1.54 (1.10-2.15)** *	1.18 (0.84-1.66)	0.241
Model 2	1.00	1.26 (0.89-1.80)	**1.58 (1.12-2.23)** *	1.22 (0.86-1.72)	0.173
C22:0					
Median, %	0.42	1.40	1.66	1.97	
Cases/n	64/141	76/142	69/142	72/142	
Model 1	1.00	1.15 (0.82-1.60)	1.15 (0.82-1.62)	1.07 (0.76-1.49)	0.752
Model 2	1.00	1.19 (0.85-1.67)	1.17 (0.83-1.65)	1.11 (0.78-1.57)	0.622
C24:0					
Median, %	3.86	4.49	5.06	6.03	
Cases/n	72/141	67/142	67/142	75/142	
Model 1	1.00	0.90 (0.65-1.26)	0.92 (0.66-1.29)	0.94 (0.68-1.30)	0.985
Model 2	1.00	0.96 (0.68-1.34)	0.92 (0.68-1.28)	0.95 (0.68-1.33)	0.733

Abbreviations: Q, quartiles; VLCSFA, very-long-chain saturated fatty acid.

[a] Cox regression analysis. Model 1: adjusted for baseline age; Model 2: further adjusted for education proportion, household income, smoking status, alcohol consumption, body mass index, hypertension, diabetes mellitus, physical activity, dietary nut intake, total energy intake, and years since menopause (women only) at baseline, as well as use of lipid-lowering medications during the whole follow-up period.

[b] Linear trends across increasing quartiles were tested by assuming that the quartiles were continuous variables.

*: P<0.05; **: P<0.01, compared with quartile 1.

Supplemental Table S4. Hazards ratios (HR) and 95% confidence interval (CI) for per standard deviation (SD) increase of the erythrocyte VLCSFA proportions [a]

Fatty acids	Mean (SD), % of total fatty acids		HR (95% CI)	P-value
	CAP cases	Non-CAP cases		
**Whole (n=2198)**				
Total VLCSFA	6.89 (1.73)	7.06 (1.70)	**0.93 (0.86-0.99)** *	**0.030**
C20:0	0.49 (0.31)	0.47 (0.17)	1.03 (0.97-1.09)	0.300
C22:0	1.47 (0.64)	1.53 (0.64)	0.96 (0.89-1.02)	0.185
C24:0	4.93 (1.24)	5.05 (1.25)	**0.92 (0.86-0.99)** *	**0.018**
**Female (n=1631)**				
Total VLCSFA	6.89 (1.75)	7.14 (1.69)	**0.88 (0.81-0.96)** **	**0.006**
C20:0	0.49 (0.23)	0.48 (0.18)	0.99 (0.89-1.11)	0.899
C22:0	1.49 (0.65)	1.58 (0.63)	**0.91 (0.84-0.99)** *	**0.021**
C24:0	4.91 (1.25)	5.07 (1.22)	**0.89 (0.82-0.97)** *	**0.011**
**Male (n=567)**				
Total VLCSFA	6.87 (1.68)	6.76 (1.70)	1.01 (0.91-1.14)	0.817
C20:0	0.48 (0.43)	0.44 (0.11)	1.05 (0.99-1.11)	0.127
C22:0	1.42 (0.62)	1.36 (0.65)	1.06 (0.94-1.19)	0.328
C24:0	4.97 (1.21)	4.96 (1.33)	0.97 (0.87-1.09)	0.629

Abbreviations: VLCSFA, very-long-chain saturated fatty acid; CAP, carotid artery plaque.

[a] Cox regression with adjustment for baseline age, gender (only in whole participants), education proportion, household income, smoking status, alcohol consumption, body mass index, hypertension, diabetes mellitus, physical activity, dietary nut intake, total energy intake, and years since menopause (women only) at baseline, as well as use of lipid-lowering medications during the whole follow-up period.

*: $P<0.05$; **: $P<0.01$, compared with quartile 1.

Supplemental Table S5. Hazards ratios (95% confidence interval) for carotid artery plaques according to quartiles of the erythrocyte VLCSFA proportions stratified by gender [a]

	Quartiles by erythrocyte VLCSFA proportions				P-trend [b]
	Q1	Q2	Q3	Q4	
**Women (n=1711)**					
Total VLCSFA					
Median, %	5.26	6.35	7.27	8.72	
Cases/n	163/429	146/430	130/429	136/430	
Model 1	1.00	0.97 (0.77-1.21)	0.99 (0.79-1.25)	**0.77 (0.61-0.97)** *	**0.037**
Model 2	1.00	0.96 (0.77-1.21)	1.01 (0.80-1.27)	0.81 (0.64-1.02)	0.104
C20:0					
Median, %	0.37	0.42	0.46	0.60	
Cases/n	148/429	142/430	145/430	140/429	
Model 1	1.00	0.91 (0.72-1.15)	0.99 (0.79-1.25)	0.82 (0.65-1.03)	0.155
Model 2	1.00	0.89 (0.71-1.13)	0.97 (0.77-1.22)	0.83 (0.66-1.05)	0.204
C22:0					
Median, %	0.47	1.52	1.76	2.09	
Cases/n	164/429	154/430	126/429	131/430	
Model 1	1.00	0.95 (0.76-1.19)	0.92 (0.73-1.17)	**0.71 (0.56-0.89)** **	**0.004**
Model 2	1.00	0.97 (0.78-1.21)	1.00 (0.78-1.27)	**0.77 (0.61-0.98)** *	**0.045**
C24:0					
Median, %	3.92	4.55	5.11	6.19	
Cases/n	151/430	141/429	150/429	133/430	
Model 1	1.00	1.01 (0.80-1.27)	1.21 (0.96-1.52)	0.85 (0.67-1.07)	0.389
Model 2	1.00	1.03 (0.81-1.29)	1.18 (0.94-1.48)	0.88 (0.69-1.11)	0.501
**Men (n=806)**					
Total VLCSFA					
Median, %	5.18	6.16	7.00	8.36	
Cases/n	96/202	87/201	98/202	98/201	
Model 1	1.00	0.82 (0.61-1.10)	1.02 (0.77-1.36)	0.85 (0.64-1.13)	0.540
Model 2	1.00	0.77 (0.57-1.03)	1.07 (0.80-1.44)	0.86 (0.65-1.15)	0.769
C20:0					
Median, %	0.35	0.39	0.43	0.54	
Cases/n	96/202	83/201	90/201	110/202	
Model 1	1.00	1.09 (0.81-1.46)	1.21 (0.91-1.62)	1.02 (0.77-1.34)	0.803
Model 2	1.00	1.13 (0.84-1.53)	1.20 (0.89-1.61)	1.06 (0.80-1.40)	0.661
C22:0					
Median, %	0.40	1.39	1.63	1.96	
Cases/n	94/202	101/201	90/202	94/201	
Model 1	1.00	0.87 (0.65-1.15)	0.85 (0.63-1.13)	0.85 (0.64-1.14)	0.300
Model 2	1.00	0.79 (0.59-1.05)	0.84 (0.63-1.14)	0.85 (0.63-1.14)	0.414
C24:0					
Median, %	3.86	4.51	5.05	5.99	
Cases/n	93/201	90/202	99/202	97/201	
Model 1	1.00	1.01 (0.75-1.35)	1.25 (0.94-1.67)	0.97 (0.73-1.29)	0.845
Model 2	1.00	1.08 (0.80-1.45)	1.32 (0.99-1.76)	1.03 (0.77-1.37)	0.569

Abbreviations: Q, quartiles; VLCSFA, very-long-chain saturated fatty acid.

[a] Cox regression analysis. Model 1: adjusted for age at the first follow up visit; Model 2: further adjusted for education proportion, household income, smoking status, alcohol consumption, body mass index, hypertension, diabetes mellitus, physical activity, dietary nut intake, total energy intake, and years since

menopause (women only) at the first follow up visit, as well as use of lipid-lowering medications during the whole follow-up period.

[b] Linear trends across increasing quartiles were tested by assuming that the quartiles were continuous variables.

*: $P<0.05$;**: $P<0.01$, compared with quartile 1.

Supplemental Table S6. Characteristics of the eligible studies.

Author (Year)	Study design, Study name or location	Duration of follow-up (y)	Total N (n cases)	Age (year), Male (%)	Source of VLCSFAs	Main outcomes	Adjusted confounders	Results	Total score of NOS
Papandreou (2019)	Nested case-control study, The PREDIMED trial	-	408 (136)	55 – 80, 59.3	Blood cell membranes	CHD (fatal and non-fatal)	Smoking and DM status	Per SD<br>C20:0 1.01 (0.81-1.25)<br>C22:0 0.44 (0.28-0.69)<br>C24:0 0.41 (0.25-0.65)<br>Total VLCSFAs 0.45 (0.29-0.70)	6
Kleber (2018)	Cohort study, The LURIC	9.9	3259 (614)	62.7 ± 10.6, 69.6	Red blood cell	CVD mortality (fatal)	Age, gender, BMI, LDL-C, HDL-C, logTG, hypertension, DM, smoking, PA, estimated glomerular filtration rate, and lipid-lowering therapy	Per SD<br>C20:0 0.97 (0.90-1.05)<br>C22:0 1.01 (0.93-1.09)<br>C24:0 1.05 (0.97-1.13)	8
Lemaitre (2018)	Cohort study, The CHS	10.6	4249 (1304)	75.1 ± 5.3, 40.4	Plasma phospholipid	Heart failure (fatal and non-fatal)	Age, sex, race, enrollment site, prevalent CHD, AF, DM, fasting glucose, treated hypertension, SBP, BMI, WC, smoking status, PA, and proportions of EPA and nervonic acid	Q5 vs Q1<br>C20:0 0.72 (0.59-0.88)<br>C22:0 0.72 (0.60-0.87)<br>C24:0 0.67 (0.55-0.81)	8
Fretts (2016)	Cohort study, The CHS	11.5	3941 (1216 and 788)	65 – 98, 41.0	Plasma phospholipid	CVD mortality and CHD mortality (fatal)	Age, sex, race, clinic, education, smoking, alcohol use, BMI, WC, PA, treated hypertension, prevalent DM, prevalent CVD, self-reported prevalent cancer, and self-reported health status at baseline	Q5 vs Q1<br>For CVD mortality<br>C20:0 0.87 (0.73-1.05)<br>C22:0 0.80 (0.66-0.96)<br>C24:0 0.72 (0.60-0.87)<br>For CHD mortality<br>C22:0 0.88 (0.70-1.11)<br>C24:0 0.81 (0.65-1.02)	8
Chung (2015)	Case-control study, Korea	-	60 (21)	45 – 87, 41.7	Plasma phospholipid	Cardioembolic stroke (fatal and non-fatal)	Age, sex, BMI, coexistence of hypertension, dyslipidemia, and DM and smoking	T3 vs T1<br>C20:0 1.79 (0.37-8.59)<br>C22:0 1.43 (0.28-7.32)<br>C24:0 0.10 (0.02-0.48)	7
Malik (2015)	Nested case-control study, The NHS	-	762 (348)	30 – 75, 0	Plasma and erythrocytes	CHD (non-fatal MI and fatal CHD)	Age at blood draw, smoking status, fasting status, and time of blood draw, BMI, PA, alcohol use, parental history of MI before age 65 years, menopausal status and hormone use, use of aspirin, the alternate Healthy Eating	Q5 vs Q1<br>In plasma<br>C20:0 0.48 (0.25-0.92)<br>C22:0 0.36 (0.19-0.69)<br>C24:0 0.41 (0.21-0.79)<br>Total VLCSFAs	7

Author (Year)	Study design, Study name or location	Duration of follow-up (y)	Total N (n cases)	Age (year), Male (%)	Source of VLCSFAs	Main outcomes	Adjusted confounders	Results	Total score of NOS
							Index score, history of hypercholesterolemia, DM, or hypertension, and erythrocyte trans FAs and long-chain n-3 FAs	0.37 (0.19-0.70)<br>In erythrocyte<br>C20:0 0.66 (0.29-1.49)<br>C22:0 0.54 (0.26-1.12)<br>C24:0 0.66 (0.30-1.44)<br>Total VLCSFAs<br>0.39 (0.18-0.86)	
Malik (2015)	Nested case-control study, The HPFS	-	1265 (446)	30 – 75, 100	Plasma and erythrocytes	CHD (non-fatal MI and fatal CHD)	Age at blood draw, smoking status, fasting status, and time of blood draw, BMI, PA, alcohol use, parental history of MI before age 65 years, menopausal status and hormone use, use of aspirin, the alternate Healthy Eating Index score, history of hypercholesterolemia, DM, or hypertension, and erythrocyte trans FAs and long-chain n-3 FAs	Q5 vs Q1<br>In plasma<br>C20:0 0.63 (0.38-1.05)<br>C22:0 0.78 (0.46-1.33)<br>C24:0 0.56 (0.33-0.97)<br>Total VLCSFAs<br>0.57 (0.34-0.96)<br>In erythrocyte<br>C20:0 0.69 (0.45-1.08)<br>C22:0 1.27 (0.76-2.12)<br>C24:0 1.09 (0.59-2.02)<br>Total VLCSFAs<br>0.89 (0.49-1.61)	7
Fretts (2014)	Cohort study, The CHS	11.2	2899 (707)	74.7 ± 5.1, 36.4	Plasma phospholipid	AF (fatal and non-fatal)	Age, sex, race, clinic, education, smoking, alcohol use, BMI, WC, PA, treated hypertension, DM, history of stroke, history of heart failure, and plasma phospholipid long-chain n-3 FAs	Q4 vs Q1<br>C20:0 0.78 (0.63-0.97)<br>C22:0 0.62 (0.50-0.78)<br>C24:0 0.68 (0.55-0.85)	7
Lemaitre (2014)	Case-control study, The United States	-	680 (265)	25 – 74, 81	Erythrocyte	Sudden cardiac arrest (fatal and non-fatal)	Age, gender, current smoking, DM, hypertension, education beyond high school, leisure-time PA, index of fat intake, body weight, height, erythrocyte proportions of EPA+DHA and trans-18:2	Per SD<br>C20:0 0.76 (0.62-0.92)<br>C22:0 0.78 (0.63-0.96)<br>C24:0 0.74 (0.60-0.92)<br>Q4 vs Q1<br>C20:0 0.42 (0.23-0.77)<br>C22:0 0.35 (0.19-0.65)<br>C24:0 0.52 (0.27-0.98)	6

Author (Year)	Study design, Study name or location	Duration of follow-up (y)	Total N (n cases)	Age (year), Male (%)	Source of VLCSFAs	Main outcomes	Adjusted confounders	Results	Total score of NOS
Matthan (2014)	WHI-OS	4.5	2488(1244)	67.8 ± 0.2, 0	Plasma phospholipid	CHD	age, enrollment date, race/ethnicity, hysterectomy status, BMI, SBP, smoking, education, medication (anticoagulant, antidiabetic, antilipid) and hormone (estrogen and/or progesterone) use, family history of cardiovascular disease/stroke/MI and type 2 DM, PA, carbohydrate, protein, and alcohol intake (percent energy)	Per SD<br>C20:0 0.99 (0.60-1.62)<br>C22:0 1.35 (0.90-2.01)<br>C24:0 1.22 (0.83-1.80)	
Matsumoto (2013)	Nested case-control study, The PHS	-	1576 (788)	58.7 ± 8.0 100	Plasma phospholipid	heart failure (non-fatal)	age, race, year of birth, the date of blood kid returned, BMI, smoking status, exercise proportion, alcohol consumption, history of DM, history of AF and all of SFAs	Per SD<br>C20:0 0.93 (0.71-1.23)<br>C22:0 0.93 (0.74-1.16)<br>Q4 vs Q1<br>C20:0 0.81 (0.41-1.59)<br>C22:0 0.86 (0.46-1.56)	7

Abbreviations: NOS, the Newcastle-Ottawa Quality Assessment Scale, which included three parts as selection, comparability and exposure or outcome. The PREDIMED trial, the Prevención con Dieta Mediterránea trial; The LURIC, the Ludwigshafen Risk and Cardiovascular Health study; The CHS, the Cardiovascular Health Study; The NHS and HPFS, the Nurses' Health Study and the Health Professionals Follow-Up Study; WHI-OS, Women's Health Initiative observational study; The PHS, the Physicians' Health Study; VLCSFA, very-long-chain saturated fatty acid; CHD, coronary heart diseases; DM, diabetes mellitus; CVD, cardiovascular diseases; BMI, body mass index; LDL-C, low-density lipoprotein cholesterol; HDL-C, high-density lipoprotein cholesterol; TG, triglyceride; WC, waist circumference; PA, physical activity; EPA, eicosapentaenoic acid; FAs, fatty acids; AF, atrial fibrillation; SBP, systolic blood pressure; MI, myocardial infarction; SFA, saturated fatty acid; DHA, docosahexaenoic Acid.

References:

1. Lemaitre, R.N.; McKnight, B.; Sotoodehnia, N.; Fretts, A.M.; Qureshi, W.T.; Song, X.; King, I.B.; Sitlani, C.M.; Siscovick, D.S.; Psaty, B.M., et al. Circulating Very Long-Chain Saturated Fatty Acids and Heart Failure: The Cardiovascular Health Study. J Am Heart Assoc **2018**, 7, e010019, doi:10.1161/JAHA.118.010019.
2. Fretts, A.M.; Mozaffarian, D.; Siscovick, D.S.; King, I.B.; McKnight, B.; Psaty, B.M.; Rimm, E.B.; Sitlani, C.; Sacks, F.M.; Song, X., et al. Associations of Plasma Phospholipid SFAs with Total and Cause-Specific Mortality in Older Adults Differ According to SFA Chain Length. J Nutr **2016**, 146, 298-305, doi:10.3945/jn.115.222117.
3. Fretts, A.M.; Mozaffarian, D.; Siscovick, D.S.; Djousse, L.; Heckbert, S.R.; King, I.B.; McKnight, B.; Sitlani, C.; Sacks, F.M.; Song, X., et al. Plasma phospholipid saturated fatty acids and incident atrial fibrillation: the Cardiovascular Health Study. J Am Heart Assoc **2014**, 3, e000889, doi:10.1161/JAHA.114.000889.
